# The pathogenesis of a North American H5N2 clade 2.3.4.4 group A highly pathogenic avian influenza virus in surf scoters (*Melanitta perspicillata*)

**DOI:** 10.1186/s12917-020-02579-x

**Published:** 2020-09-23

**Authors:** Jasmina M. Luczo, Diann J. Prosser, Mary J. Pantin-Jackwood, Alicia M. Berlin, Erica Spackman

**Affiliations:** 1 Department of Agriculture-Agricultural Research Service, Southeast Poultry Research Laboratory, U.S. National Poultry Research Center, U.S., 934 College Station Road, Athens, GA 30605 USA; 2 US Geological Survey, Patuxent Wildlife Research Center, 12100 Beech Forest Road, Laurel, MD 20708 USA

**Keywords:** Highly pathogenic avian influenza virus, Pathogenesis, Diving duck, Surf scoter, H5Nx

## Abstract

**Background:**

Aquatic waterfowl, particularly those in the order *Anseriformes* and *Charadriiformes*, are the ecological reservoir of avian influenza viruses (AIVs). Dabbling ducks play a recognized role in the maintenance and transmission of AIVs. Furthermore, the pathogenesis of highly pathogenic AIV (HPAIV) in dabbling ducks is well characterized. In contrast, the role of diving ducks in HPAIV maintenance and transmission remains unclear. In this study, the pathogenesis of a North American A/Goose/1/Guangdong/96-lineage clade 2.3.4.4 group A H5N2 HPAIV, A/Northern pintail/Washington/40964/2014, in diving sea ducks (surf scoters, *Melanitta perspicillata*) was characterized.

**Results:**

Intrachoanal inoculation of surf scoters with A/Northern pintail/Washington/40964/2014 (H5N2) HPAIV induced mild transient clinical disease whilst concomitantly shedding high virus titers for up to 10 days post-inoculation (dpi), particularly from the oropharyngeal route. Virus shedding, albeit at low levels, continued to be detected up to 14 dpi. Two aged ducks that succumbed to HPAIV infection had pathological evidence for co-infection with duck enteritis virus, which was confirmed by molecular approaches. Abundant HPAIV antigen was observed in visceral and central nervous system organs and was associated with histopathological lesions.

**Conclusions:**

Collectively, surf scoters, are susceptible to HPAIV infection and excrete high titers of HPAIV from the respiratory and cloacal tracts whilst being asymptomatic. The susceptibility of diving sea ducks to H5 HPAIV highlights the need for additional research and surveillance to further understand the contribution of diving ducks to HPAIV ecology.

## Background

Avian influenza viruses (AIVs) are ubiquitous in aquatic birds [1], particularly waterfowl, shorebirds, and gulls in the orders *Anseriformes* and *Charadriiformes*. AIVs are classified as low or high pathogenicity based on disease characteristics in gallinaceous poultry, or molecular attributes of the influenza surface glycoprotein, hemagglutinin [2]. Of global public and animal health concern is the A/goose/Guangdong/1/1996 (Gs/Gd/96)-lineage of H5 highly pathogenic AIVs (HPAIVs). Infection of poultry with Gs/Gd/96-lineage H5 HPAIVs is associated with high morbidity and mortality rates, and all laboratory-confirmed human H5 HPAIV infections to date are associated with this H5 lineage [3]. Furthermore, some aquatic bird species infected with most Gs/Gd/96-lineage HPAIVs have been shown to be asymptomatic whilst shedding HPAIV [4–9]. This unique characteristic of HPAIV infection dynamics in aquatic birds has likely facilitated the global movement of HPAIVs in migratory birds. In North America, there are four major wild bird migratory routes: the Pacific, Mississippi, Atlantic, and Central flyways. Migratory wild birds within these flyways maintain a diverse AIV gene pool, facilitating the maintenance, spread and diversification of AIV populations [10]. Globally, the North American Pacific flyway overlaps the West Pacific flyway, which itself overlaps the East Asian-Australasian flyway that encompasses China, Korea and much of Southeast Asia. This global flyway connection eventuated in the first detection of the Gs/Gd/96-lineage H5 HPAIV in North America in 2014 [11].

Within the *Anseriformes* order, non-goose waterfowl can be characterized as dabbling ducks or diving ducks based on foraging behaviours and locales. Dabbling ducks forage at the water surface where AIVs are known to persist [12, 13], facilitating fecal-oral transmission. Consequently, dabbling ducks serve as a major natural reservoir of AIVs and surveillance efforts have focused primarily on monitoring AIV populations in dabbling ducks [14]. In contrast, diving ducks forage by diving and swimming underwater. It is increasingly recognized that diving ducks play an underappreciated role in the maintenance and transmission of AIVs, leading to increased research on the contribution of these species to the ecology and evolution of AIVs [15–17]. Diving ducks have been shown to harbor rare AIVs subtypes [18], and diving ducks contributed to the emergence of North American H7 HPAIV [19]. Further, experimentally infected diving ducks can shed HPAIV in the absence of clinical disease signs [20–23], and may contribute to the spread of HPAIV. Nevertheless, there remains a paucity of information on the role diving ducks play in the maintenance and spread of HPAIVs. Moreover, the susceptibility to, and disease dynamics of emerging HPAIVs in diving ducks remains understudied.

Surf scoters are large diving sea ducks native to North America, with breeding grounds situated in the boreal tundra transitional zone across northern Canada and Alaska, and wintering areas located on both the east and west coasts of continental USA [24]. The geographical distribution of surf scoters overlaps that of migratory birds known to transmit HPAIVs. Consequently, it is important to understand the potential role of surf scoters in HPAIV spread and evolution. In this study, the pathogenesis of a clade 2.3.4.4 H5 HPAIV in surf scoters was characterized. Surf scoters were experimentally infected via the intrachoanal route with a North American Gs/Gd/96-lineage clade 2.3.4.4 group A H5N2 HPAIV, and clinical disease, histopathology, and virus shedding are described.

## Results

### Clinical disease

Surf scoters were inoculated intrachoanally with 1 × 10^6^ 50% egg infectious doses (EID_50_) of A/Northern Pintail/Washington/40964/2014 (NP/WA/14) H5N2 HPAIV and were monitored daily for clinical disease signs. Overall, surf scoters inoculated with NP/WA/14 were generally asymptomatic, exhibiting no or very mild clinical disease signs. Transient mild lethargy was observed at 5 days post inoculation (dpi), though ducks responded immediately upon stimulation. Cloacal (CL) temperatures were recorded throughout the duration of the study and were within the expected normal range for ducks: average CL temperatures were 40.4 °C, 41.1 °C, 40.8 °C, and 40.4 °C at 0, 2, 4, and 7 dpi, respectively (Additional file 1A). When expressed as percent starting temperature, average temperature throughout the duration of the study did not exceed 101.7% (range: 97.4–104.5%) (Additional file 1B). As all CL temperatures recorded were < 42.0 °C, it was therefore considered that HPAIV infection did not induce fever in surf scoters. Despite all ducks exhibiting transient mild clinical disease signs at 5 dpi only, two aged birds died during the study (both were 16 years of age). One aged bird died 5 dpi (# 629), and the other aged bird died 14 dpi (# 635). An empty digestive tract was noted for the bird that died at 5 dpi. Gross lesions observed during necropsy of bird # 629 included a minor esophageal fungal lesion, a fixed pupil, and corneal edema. Duck 635 that died 14 dpi had an enlarged hard spleen and chronic *Candida albicans*. No other gross lesions were observed in the surviving birds.

### Serum antibody response

The influenza seronegative status of all birds was verified by anti-influenza A nucleoprotein (NP) blocking enzyme-linked immunosorbent assay (ELISA) prior to challenge (Table 1). At 14 dpi, all surviving ducks were bled for serum antibody analysis. All ducks seroconverted following challenge with NP/WA/14. Post-challenge serum hemagglutinin inhibition (HI) titers ranged from 2^5^ to 2^9^, with the geometric mean being 2^6.9^ (95% confidence interval (CI): 2^5.8^–2^8.2^) (Table 1). Post-challenge anti-influenza A NP ELISA results agreed with HI results, all ducks were positive for anti-influenza A NP IgY antibodies (Table 1).

**Table 1:** Serostatus of surf scoters pre- and post-inoculation with H5 HPAIV NP/WA/14 (H5N2). Whole blood was collected 14 dpi and serum was separated for antibody analysis

Bird age/sex^a^	Pre-challenge ELISA^b^	Post-challenge ELISA^b^	Log_2_ serum HAI titer
2/M	neg	pos	7
4/M	neg	pos	6
4/M	neg	pos	8
5/M	neg	pos	9
6/M	neg	pos	5
6/M	neg	pos	7
7/M	neg	pos	7
16/M	neg	nd	nd
16/F	neg	nd	nd

Anti-influenza A nucleoprotein IgY response was determined by blocking ELISA using commercial kit. Serum hemagglutinin inhibition titers were determined according to the standard USDA HI procedure (Pedersen et al. 2014)

^a^Age reported in years. M = male, F = female

^b^neg = seronegative for anti-influenza A NP IgY serum antibodies

*Pos* Seropositive for anti-influenza A NP IgY serum antibodies, *Nd* Not done, the birds had died

### Virus shedding

**Fig. 1:**
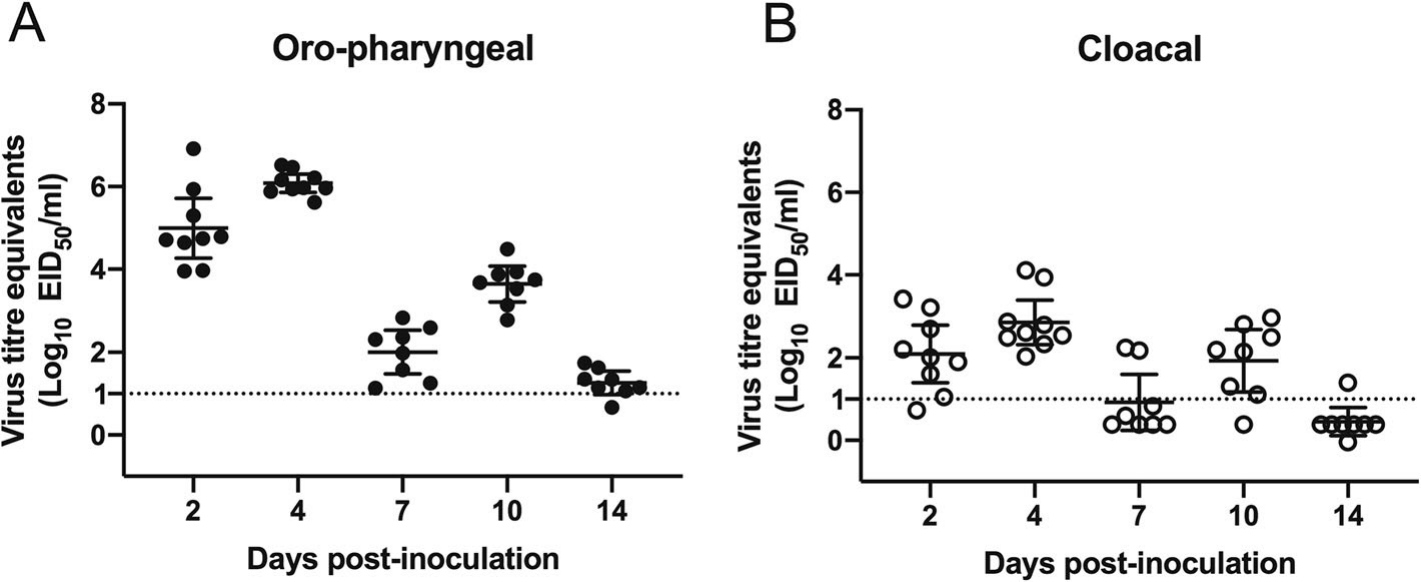
Virus titer equivalents in swabs collected from A/Northern pintail/Washington/40964/2014 (H5N2) inoculated surf scoters (*n* = 9) at 2, 4, 7, 10, and 14 dpi and detected by real-time qRT-PCR. **a** Oropharyngeal swabs. **b** Cloacal swabs. The dotted line indicates limit of detection. Error bars represent mean ± 95% CI

Shedding from oropharyngeal (OP) and CL routes of surf scoters inoculated with clade 2.3.4.4 NP/WA/14 (H5N2) HPAIV was examined. Virus titer equivalents were determined 2, 4, 7, 10, and 14 dpi by real-time qRT-PCR. The peak of HPAIV shed from the OP tract was at 4 dpi, and HPAIV shed from the CL tract also peaked at 4dpi. Mean virus titer equivalents detected in OP and CL swabs at 4 dpi were 10^6.1^ and 10^2.9^, respectively. Overall, a bimodal shedding pattern was observed for OP and CL shedding, with virus titer equivalent peaks occurring at 4 dpi, then again at 10 dpi. Virus titer equivalents detected in the second peak at 10 dpi (OP, 10^3.7^; CL, 10^1.9^) were lower than virus titer equivalents detected in the first peak. This shedding pattern may be due to reinfection, or by initial production of type I interferons (bimodal shedding patterns in humans have been attributed to type I interferon production [25]) followed by the induction of humoral immunity as the infection progressed. Virus was shed from the OP route up until 10 dpi, and only some birds shed low-levels of virus at 14 dpi. Moderate virus titer equivalents were shed from the CL route, with a pattern of shedding following that of OP. Higher virus titer equivalents were detected in OP swabs at each time point throughout the duration of the study (Fig. 1; Additional file 2A). Additionally, area under the curve analysis revealed that overall shedding from the OP route was significantly higher than from the CL route (Additional file 2B), a finding consistent with previous reports characterizing HPAIV shedding patterns from experimentally infected ducks [23, 26–28].

### Histopathology and immunohistochemistry

**Fig. 2:**
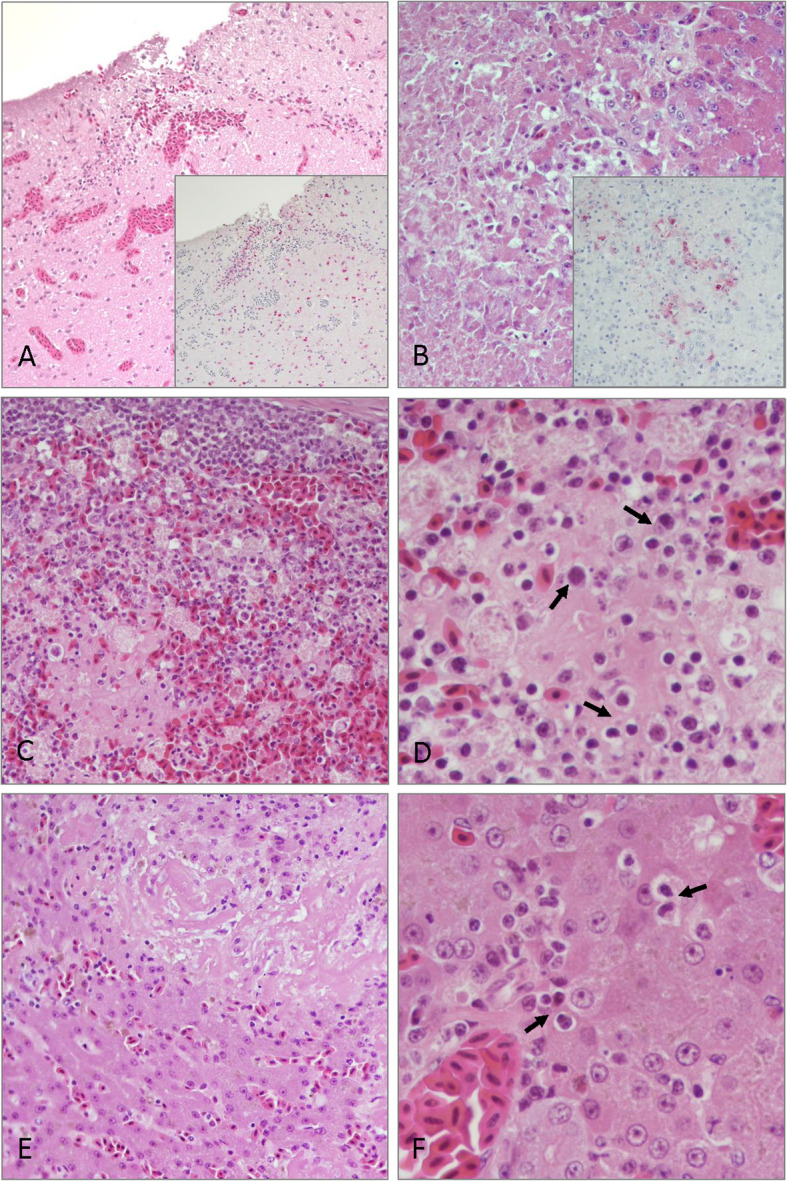
Histopathology and immunohistochemical detection of influenza virus antigen in tissues from Surf Scoters inoculated with A/Northern pintail/Washington/40964/2014 (H5N2) HPAIV. **a** Cerebrum. Congestion and necrosis. Inset: virus antigen in neurons and glial cells (red staining), 20X magnification. **b** Pancreas. Acinar cell degeneration and necrosis. Inset: virus antigen in acinar cells and mononuclear cells (red staining), 40X magnification. **c** Spleen. Parenchymal cell degeneration and necrosis with accumulation of proteinaceous, fibrin-like material, 40X magnification. **d** Spleen. Presence of basophilic intranuclear inclusion bodies in reticular endothelial cells (arrows), 60X magnification. **e** Liver. Hepatocyte degeneration and necrosis and replacement with proteinaceous, fibrin-like material. **f** Liver. Presence of basophilic intranuclear inclusion bodies in hepatocytes (arrows), 60X magnification

The ducks that died were the two oldest ducks in the group (16 years). One duck died at 5 dpi (# 629, male) and the other at 14 dpi (# 635 female). Duck 629 had empty intestines at necropsy. Duck 635 had an enlarged hard spleen and chronic *Candida albicans*. On histopathologic examination, both ducks presented similar microscopic lesions. These lesions included moderate to severe multifocal necrosis in the liver, spleen, and pancreas, with presence of basophilic intranuclear and cytoplasmic inclusion bodies in degenerating and perilesional cells (Fig. 2, Additional file 3). Parenchymal cell apoptosis, coagulative necrosis, and serofibrinous and haemorrhagic lesions were present in all these organs. Also, in both ducks, there was mild-to-moderate diffuse tracheitis, enteritis, and mild interstitial pneumonia. In duck 629 there was also multifocal areas of congestion and necrosis in the cerebrum. In this duck, viral antigen was present in neurons and glial cells of the brain, in pancreatic acinar cells (Fig. 2), in the epithelial cells and infiltrating mononuclear cells in the trachea, in cardiomyocytes, and proventricular gland epithelium (Additional file 3). No avian influenza viral antigen was present in tissues collected from duck 635.

The presence of widespread inclusion bodies in the spleen, liver, and pancreas in addition to marked fibrinous-like deposits associated with necrotic lesions in the same tissues suggested that the ducks that succumbed to infection may have been concurrently infected with the herpesvirus, duck enteritis virus (DEV) (*Anatid herpesvirus 1*). DEV exhibits a tropism for epithelial cells of the gastrointestinal tract, and can be detected in CL swab fluid [29]. To confirm the histological findings, a DEV specific PCR test was conducted with nucleic acid extracted from all the CL swabs from all the ducks. Duck 635 was positive for DEV at 7 and 10 dpi, whilst DEV was not detected in CL swab fluid from duck 629. However, DEV was detected in duck 482 CL swab fluid at 4 dpi. Duck 482 was a 6-year-old duck that did not exhibit any clinical signs of DEV infection.

## Discussion

The Gs/Gd/96-lineage of H5 HPAIVs has undergone extensive genetic divergence, leading to the formation of multiple genetic clades and subclades [30]. A unique attribute of the currently circulating clade 2.3.4.4 H5Nx HPAIVs is the compatibility of the HA gene with multiple neuraminidase subtypes, including N2, N6, and N8 [31, 32]. Novel clade 2.3.4.4 H5Nx HPAIVs were initially detected in China in 2008 [30, 33, 34], followed by outbreaks in South Korea then Japan in 2014 [35–37]. Radiating outwards from Asia, H5Nx HPAIVs rapidly spread globally and were detected in Russia [38], Germany [39], England [40], the Netherlands, Hungary and Italy [41, 42]. The Gs/Gd/96-lineage H5Nx HPAIVs were first detected in Canada and North America in 2014 [11, 43]. Whilst one index virus from the North American Gs/Gd/96-lineage 2014 HPAIV outbreak, A/gyrfalcon/Washington/41088–6/2014 (H5N8), is a wholly Eurasian H5Nx virus, the NP/WA/14 (H5N2) HPAIV characterized in this study is of mixed origin, with evidence for reassortment with North American low pathogenicity avian influenza viruses [44]. Although H5N8 HPAIVs continue to be detected in wild bird populations globally, the NP/WA/14 isolate is of interest because the related H5N2 lineage became the predominant lineage in North America. It was the most common of the 4 clade 2.3.4.4 variants detected in domestic birds and was the only variant identified in North American surveillance samples from wild birds after the outbreak [45], although it has not been detected in North America since 2016. However, sporadic outbreaks of a related H5N2 reassortants continue to be detected in poultry in Taiwan [46]. Therefore, there may be some fitness advantage to this gene constellation in domestic birds.

Wild aquatic waterfowl likely played a major role in the dissemination of these novel H5Nx viruses in the 2014–2015 North American outbreak [31, 47, 48]. Importantly, to understand the capacity for HPAIV replication in migratory waterfowl and their potential role in H5Nx global dissemination, it is vital to understand the pathogenesis of H5Nx HPAIV in diving ducks to complement what is known for dabbling ducks.

To assess the susceptibility to, and pathogenesis of the novel North American Gs/Gd/96-lineage H5 HPAIV in diving ducks, *Melanitta perspicillata*, were inoculated by the intrachoanal route and clinical disease and virus excretion were evaluated. Inoculation of surf scoters with North American group A H5Nx HPAIV induced minimal disease signs despite ducks shedding high titers of virus, particularly from the OP route. The propensity for H5 HPAIV-infected waterfowl to be subclinical, or display mild clinical signs has been widely described in dabbling ducks [4–9, 49], with increasing research characterizing clinical disease in diving ducks. Previously, it was shown that the North American diving duck species, redhead (*Aythya americana*), are susceptible to H5 HPAIV infection [23], however, HPAIV-infected redhead did not exhibit clinical disease signs, and HPAIV was shed from the OP and CL routes for up to 4 days post-challenge. Active HPAIV shedding in the absence of clinical disease has also been reported for Lesser scaup (*Aythya affinis*) and Ruddy ducks (*Oxyura jamaicansis*), two additional North American diving duck species [20, 21]. Ruddy ducks were challenged with the same North American clade 2.3.4.4 group A HPAIV used in this study, NP/WA/14 (H5N2), whilst the other diving duck species, Lesser scaup, were challenged with either NP/WA/14 (H5N2), or the wholly Eurasian clade 2.3.4.4 group A isolate detected during the North American H5Nx outbreak, A/gyrfalcon/Washington/41088–6/2014 (H5N8). Both North American group A Gs/Gd/96-lineage H5Nx HPAIVs did not induce clinical disease signs in inoculated diving ducks, however, both species of diving ducks excreted virus for up to 7 dpi. In a subsequent study, Lesser scaup infected with H7 HPAIVs also did not display clinical disease signs, despite high levels of virus excretion from both the OP and CL routes for up to 7 and 14 dpi, respectively [21]. Peak virus titer equivalents detected in OP or CL swabs from HPAIV-infected Ruddy ducks and Lesser scaups was approximately 10^4^ EID_50_/ml, whereas shedding from HPAIV-infected surf scoters peaked at 10^6^ EID_50_/ml, highlighting some variation in shedding dynamics between various diving duck species. Low pathogenicity avian influenza viruses replicate primarily in the gastrointestinal tract leading excretion of high virus titers via the CL route [50, 51], facilitating fecal-oral transmission. In contrast, higher virus titers are typically excreted from the OP route of Gs/Gd/96-lineage-infected aquatic waterfowl and poultry [23, 26]. Surf scoters challenged with clade 2.3.4.4 group A H5Nx HPAIV shed significantly higher virus titer equivalents from the OP route compared to the CL route. In terms of transmission, the importance of higher virus titers excreted from the respiratory route of Gs/Gd/96-lineage HPAIV infected poultry and wild waterfowl is unclear, but is likely that oral secretions may be able to contaminate the environment similarly to cloacal secretions. Although likely dependent on the challenge virus, a meta analyses of virus titer shed and duration of shedding from HPAIV-infected (dabbling) ducks reported that dabbling ducks shed approximately 10^6^ EID_50_ virus from the OP route and 10^3^ EID_50_ from the CL route [52] and median shedding duration of HPAIV-infected ducks was reported to be 5 dpi [53]. Furthermore, virus excretion from experimentally inoculated domestic ducks with a panel of HPAIVs suggested that shedding duration from HPAIV-infected dabbling ducks can extend for at least 7 dpi and up to 14 dpi in some instances [27]. Shedding patterns from HPAIV-infected diving ducks appears to be for similar to that reported for dabbling ducks; surf scoters inoculated with H5N2 clade 2.3.4.4 group A HPAIV shed similar virus titers, and for a similar duration to that reported for dabbling ducks, with virus excretion peaking at approximately 10^6^ EID_50_/ml, and virus shedding detected for up to 14 dpi. Collectively, minimizing disease impact on the host, whilst maintaining the ability to be excreted at high titers from OP and CL routes favors diving ducks as additional vectors of HPAIV transmission in the wild. Further studies characterizing the pathogenesis of clade 2.3.4.4 groups B-D in surf scoters would shed light on whether surf scoters generally shed high titers of clade 2.3.4.4 HPAIVs in the absence of clinical disease signs, or whether HPAIVs in these groups induce different disease characteristics in surf scoters.

In addition to experimental infection of North American diving ducks, experimental infection of European wild diving ducks (common pochard, *Aythya ferina*) with a clade 2.3.4.4 group A H5 HPAIV also resulted in subclinical disease whilst excreting high virus titers [22]. However, subclinical disease presentation in common pochards may be clade or even subclade-dependent, as experimental infection with clade 2.2.1 HPAIV resulted in 50% mortality [8]. Recognizing the susceptibility and potential transmission of HPAIVs by wild birds, diving ducks in the genera *Aythya* and *Netta* have been identified by the European Union as high risk wild bird species for H5 infection and transmission [54].

In contrast, tufted ducks (*Aythya fuligula*), a diving duck that crosses to North America from Asia and Europe via vagrant migrants [55], naturally infected with H5N1 HPAIV seem to be highly susceptible to severe infection outcomes. High mortality was noted, and histopathological analysis of tissues from infected ducks revealed abundant viral antigen in multiple visceral and central nervous system organs [56]. Whilst European clade 2.3.4.4 group A HPAIVs caused low mortality in domestic (dabbling) ducks [40], to date, infection studies with tufted ducks have not been performed. However, naturally occurring HPAIV infection of wild tufted ducks lead to high mortality during the clade 2.3.4.4 group B HPAIV outbreak in the Netherlands in 2016/17 [57, 58]. Experimental infection of tufted ducks with a clade 2.3.2 HPAIV ultimately yielded the same disease outcomes [59]. Evidence suggests that this species of diving duck may not serve in the dispersal of HPAIVs in the absence of clinical disease [59]. It is unclear whether, genetic background of tufted ducks predisposes this species of diving duck to severe disease upon infection with H5 HPAIV, a factor demonstrated to influence influenza disease outcomes in genetically diverse mice [60, 61], or whether concomitant infection with other pathogens influences mortality outcomes in the wild tufted ducks.

In this study, pathological evidence for concomitant infection of some surf scoters with DEV was observed and confirmed with molecular techniques. Ducks in the order *Anseriformes* are susceptible to infection with DEV at all ages, though DEV-related mortality is significantly higher in mature than immature ducks [29, 62, 63]. Indeed, it was the two oldest birds that died, and DEV could have been a contributing factor. Whilst it is tempting to speculate about age-related mortality observed in this study, we exercise caution in doing so due to the small sample size. Furthermore, it is unclear whether mortality observed was directly attributed to HPAIV infection, modulated by concomitant DEV infection, or a synergism between the two viruses yielded mortality outcomes in aged scurf scoters. It is also unknown whether the concomitant DEV was an acute infection, or reactivation of latent DEV infection. Indeed, one of the aged surf scoters that died had chronic corneal edema and a fixed pupil, which can possibly be attributed to latent DEV infection since the trigeminal ganglion is a known latency site for DEV [64]. Whilst the detection of DEV was unexpected, the situation likely reflects pathogen dynamics occurring the field. Also, most of the ducks had a chronic *Candida albicans* infection, which is a topical fungal infection in the oral cavity. Because this condition is localized it’s not expected to have an appreciable affect in HPAIV pathogenesis. Ducks with other health conditions are probably more relevant to the field versus specific pathogen free animals because it is probable that the interplay of multiple pathogens and/or the physiological stress of chronic health conditions influences HPAIV disease outcomes in wild aquatic birds.

## Conclusions

Collectively, H5 HPAIV infection of surf scoters generally lead to subclinical infection whilst concomitantly excreting high titers of virus, particularly from the OP route. The capacity for HPAIVs to be shed from infected diving ducks for several days post-challenge, in the absence of clinical disease signs, likely facilitates the maintenance and potentially the long-range movement of HPAIV viruses, ultimately influencing HPAIV ecology. To further understand the emerging role of diving ducks in HPAIV transmission and ecology, HPAIV infection dynamics in diving ducks warrants further study.

## Methods

### Virus

A clade 2.3.4.4 group A H5Nx HPAIV, A/Northern pintail/Washington/40964/2014 (NP/WA/14) (H5N2), (NCBI:txid 1,589,662; genetic characterization reported by *Ip* et al. [43]) was selected for characterization in surf scoters. Although this is not a recent isolate, this isolate was selected because it has been characterized in numerous avian species [20, 27, 28, 65, 66], which allows for comparison and may contribute to an understanding of why this isolate became dominant in North America. Virus stock was generated by allantoic inoculation of 9–11 day old embryonating chicken eggs. Infectious allantoic fluid was harvested, clarified by centrifugation, and stored at − 80 °C. Virus titer was determined by titration in 9–11 day old embryonating chicken eggs, and the EID_50_/ml was calculated by the Reed and Muench method [67].

### Animals and study design

Adult surf scoters (*Melanitta perspicillata*) (*n* = 9) were sourced from U.S. Geological Survey Patuxent Wildlife Research Center captive breeding colonies (Laurel, MD, USA). All animal research was reviewed and approved by the U.S. National Poultry Research Center Institutional Animal Care and Use Committee. Scoter captivity and transport to Southeast Poultry Research Laboratory was approved by the U.S. Geological Survey Patuxent Wildlife Research Center Animal Care and Use Committee. Animal studies adhered to the Federation of Animal Science Societies Guide for the Care and Use of Agricultural Animals in Research and Training. Institutional review board review does not apply because the ducks are owned by the US government and approval by the IACUC is considered consent to use the ducks.

No sample size calculations were conducted because there were only 9 ducks available, so groups sizes could not be adjusted. However a group size of around 10 animals is consistent with numerous previous studies evaluating the pathogenesis of avian influenza virus in avian species [27, 68]). When the ducks were acquired, some had a pre-existing, laboratory confirmed (by culture) topical infection of *Candida albicans* on their bills.

Adult surf scoter age ranged from 2 to 16 years old (Table 1), and were of mixed sex (male, *n* = 8; female, *n* = 1). Ducks were housed in modified Horsfall isolators, with single pass air ventilation, HEPA filtered exhaust, grate floors and automatically filling cup drinkers. The lighting schedule was 8 h dark and 16 h light. Food and water were available ad libitum.

Surf scoters were acclimated for 1 week, during which time pre-challenge swab and blood samples were collected. Surf scoters were inoculated with 1 × 10^6^ EID_50_ of NP/WA/14 (H5N2) HPAIV via the intrachoanal route and were monitored twice daily for clinical disease signs. OP and CL swabs were collected 2, 4, 7, 10, and 14 days dpi and placed in 1 ml brain heart infusion broth supplemented with 10,000 U/ml penicillin (Sigma Aldrich; St. Louis, MO), 1 mg/ml gentamicin (Sigma Aldrich), and 20 μg/ml amphotericin B (Sigma Aldrich). Cloacal temperatures were recorded 0, 2, 4, and 7 dpi. All surviving birds were euthanized 14 dpi, at which time whole blood was collected for serum antibody analysis.

Due to the small sample size, only those birds that died during the study were necropsied for histopathological examination. Tissues collected during necropsy were placed in 10% neutral buffered formalin. Formalin-fixed tissue samples were processed according to routine histological methods, embedded in paraffin wax, sectioned (4 μm), and mounted on charged glass slides. Deparaffinized, serial tissue sections were stained with hematoxylin and eosin for histological examination or stained by immunohistochemistry using a Southeast Poultry Research Laboratory in-house anti-influenza NP monoclonal antibody (clone P13C11) to determine the tissue distribution of influenza NP antigen. Deparaffinization, antigen retrieval, blocking of endogenous peroxidases, and anti-influenza A NP immunostaining was performed as previously described [68, 69].

All surviving birds at the termination of the trial were euthanized and necropsied to evaluate gross lesions; because no lesions were observed, no tissues were collected for microscopic evaluation. Humane euthanasia of birds was performed according to American Veterinary Medical Association (AVMA) guidelines by intravenous injection of 0.2 mL per Kg of pentobarbiotal sodium solution (Fatal-Plus, Vortech Pharmaceuticals, Dearborn, MI) without sedation. This method was utilized because it was the AVMA recommended method which was reviewed and approved by the USNPRC IACUC.

### Serology

The presence of anti-influenza A NP antibodies was determined by Avian influenza MultiS-Screen commercial ELISA (IDEXX; Westbrook, ME), according to the manufacturers’ instructions. Serum from all ducks was tested prior to challenge and at 14 days post challenge.

### Hemagglutination inhibition assay

To assess serum anti-influenza hemagglutinin antibody responses, a HI assay was performed as described previously [70]. Briefly, sera were diluted 2-fold before the addition of 4 hemagglutination units and incubated at room temperature for 1 h. Chicken erythrocytes (0.5%) were then added and hemagglutination inhibition recorded. End point titers are reported as the log_2_ dilution of the last HI positive serum dilution. A titer of 16 or above was considered positive.

### RNA extraction and real-time quantitative reverse transcription PCR to determine virus titer equivalents

Virus titer equivalents in OP and CL swab fluid were determined by real-time quantitative reverse transcription PCR (qRT-PCR). RNA was extracted from OP and CL swab media using MagMAX-96 AI/ND Viral RNA Isolation Kit (Thermo Fisher; Waltham, MA) as previously described [71]. A U.S. Department of Agriculture standardized one-step real-time qRT-PCR assay targeting the influenza A matrix gene was performed as previously described [72]. To calculate virus titer equivalents, a standard curve was generated from a dilution series of the same NP/WA/14 stock (with known EID_50_/ml) used as the challenge virus.

### PCR for duck enteritis virus

To verify histological findings in aged surf scoters that died and to determine whether other ducks may have been infected, PCR amplification of the duck enteritis virus (DEV) (*Herpesviridae*; *Anatid alphaherpesvirus 1*) UL-31 gene was performed on nucleic acid extracted from all CL swabs as described above. The PCR procedure and two sets of primers (DVE-5 and DVE-7) described by Hansen et al. [73] were utilized.

### Statistical analysis

Statistical analysis was performed using Prism, version 8.3.0 (GraphPad Software, Inc., La Jolla, CA, USA). Total virus titer equivalents shed from the OP and CL routes throughout the duration of the study were compared by a mixed-effects analysis followed by Sidak’s multiple comparisons post-test. Area under the curves were compared using an unpaired *t*-test. Error bars represent mean ± 95% CIs.

## Supplementary information

**Additional file 1. **Cloacal temperatures of A/Northern pintail/Washington/40964/2014 (H5N2) inoculated surf scoters (*n* = 9). A) Cloacal temperatures at 0, 2, 4, and 7 dpi. B) Cloacal temperatures expressed as percent starting temperature.

**Additional file 2. **Comparison of virus titer equivalents shed from A/Northern pintail/Washington/40964/2014 (H5N2) HPAIV inoculated surf scoters (*n* = 9). A) Mean virus titer equivalents detected by real-time qRT-PCR in OP (closed circles) and CL (open circles) swabs collected from NP/WA/14 (H5N2) inoculated surf scoters at 2, 4, 7, 10, and 14 dpi. B) Area under the curve analyses of total virus equivalents shed from OP and CL routes throughout the duration of the study. Error bars represent mean ± 95% CI. ****p < 0.0001, **p < 0.01, *p < 0.05.

**Additional file 3.** Microscopic lesions and viral antigen distribution in tissues from surf scoters intrachoanally inoculated with A/Northern pintail/Washington/40964/2014 (H5N2) and sampled at 5 (bird #629) and 12 dpi (bird #635).

## Data Availability

The datasets used and/or analysed during the current study are available from the corresponding author on reasonable request.

## Abbreviations

AIV: Avian influenza virus
CI: Confidence intervals
CL: Cloacal
DEV: Duck enteritis virus
dpi: Days post inoculation
EID_50_: 50% egg infectious dose
ELISA: Enzyme-linked immunosorbent assay
Gs/Gd/96: A/goose/Guangdong/1/1996
HI: Hemagglutination inhibition
HPAIV: Highly pathogenic avian influenza virus
NP: Nucleoprotein
NP/WA/14: A/Northern Pintail/Washington/40964/2014
OP: Oropharyngeal
qRT-PCR: Quantitative reverse transcription PCR
