# Supplementary material for: The pathogenesis of a North American H5N2 clade 2.3.4.4 group A highly pathogenic avian influenza virus in surf scoters (Melanitta perspicillata)
Source: BMC Vet Res. 2020 Sep 23;16:351. doi: 10.1186/s12917-020-02579-x (PMC7513502; doi:10.1186/s12917-020-02579-x)
Supplement: Supplementary file 1 — Additional file 1. Cloacal temperatures of A/Northern pintail/Washington/40964/2014 (H5N2) inoculated surf scoters (n = 9). A) Cloacal temperatures at 0, 2, 4, and 7 dpi. B) Cloacal temperatures expressed as percent starting temperature. [file 12917_2020_2579_MOESM1_ESM.docx]

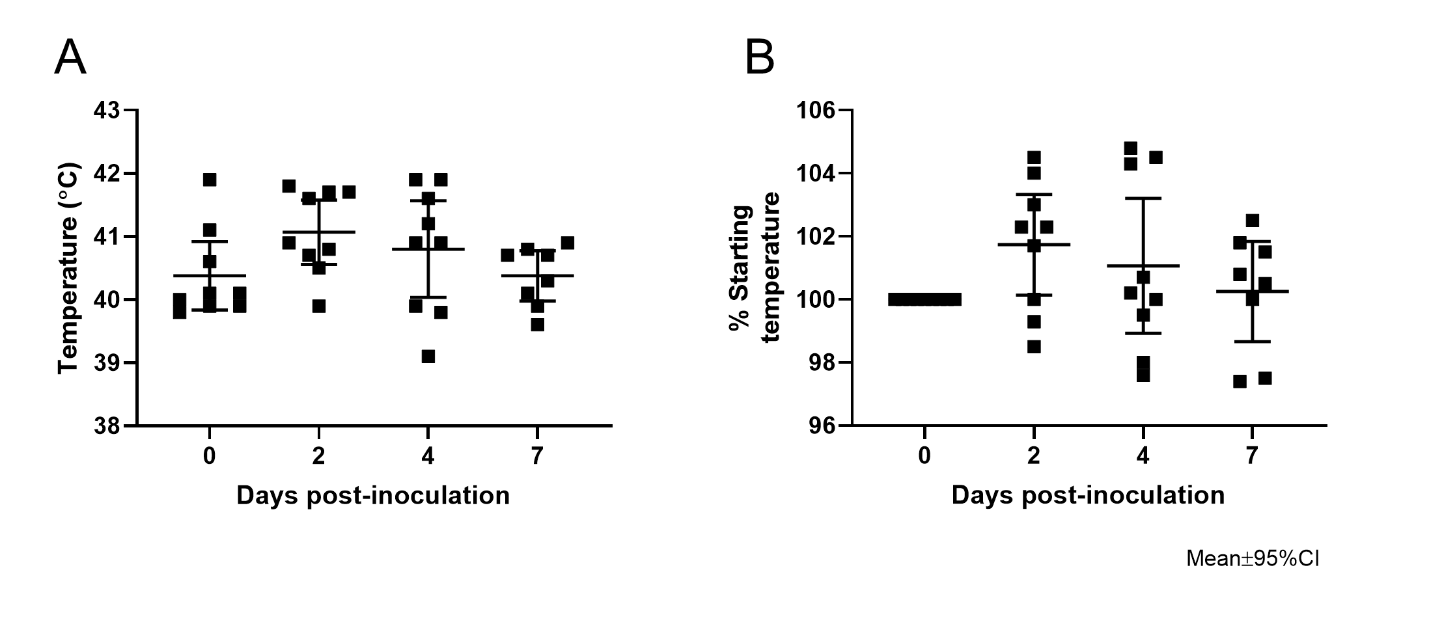


Additional File 1: Cloacal temperature of Surf scoters inoculated with A/Northern pintail/Washington/40964/2014 (H5N2) HPAIV. A). Cloacal temperature of Surf scoters 0, 2, 4, and 7 dpi following inoculation with clade 2.3.4.4 NP/WA/14 (H5N2) HPAIV (°C). B) Cloacal temperatures expressed as percent starting temperature. Days 0, 2 and 4 post inoculation n=9, day 7 post inoculation n=8. Dotted line indicates limit of detection. Error bars represent mean±95% CI.
