# Supplementary material for: The pathogenesis of a North American H5N2 clade 2.3.4.4 group A highly pathogenic avian influenza virus in surf scoters (Melanitta perspicillata)
Source: BMC Vet Res. 2020 Sep 23;16:351. doi: 10.1186/s12917-020-02579-x (PMC7513502; doi:10.1186/s12917-020-02579-x)
Supplement: Supplementary file 2 — Additional file 2. Comparison of virus titer equivalents shed from A/Northern pintail/Washington/40964/2014 (H5N2) HPAIV inoculated surf scoters (n = 9). A) Mean virus titer equivalents detected by real-time qRT-PCR in OP (closed circles) and CL (open circles) swabs collected from NP/WA/14 (H5N2) inoculated surf scoters at 2, 4, 7, 10, and 14 dpi. B) Area under the curve analyses of total virus equivalents shed from OP and CL routes throughout the duration of the study. Error bars represent mean ± 95% CI. ****p < 0.0001, **p < 0.01, *p < 0.05. [file 12917_2020_2579_MOESM2_ESM.docx]

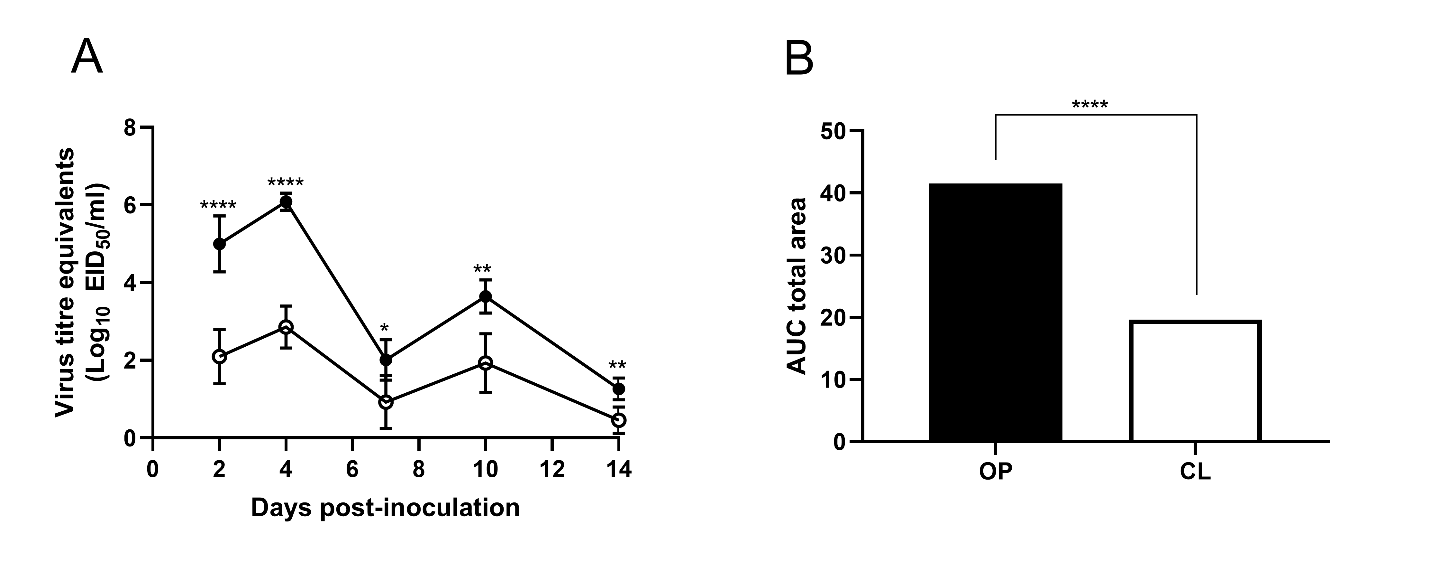


Additional File 2: Comparison of virus titer equivalents shed from A/Northern pintail/Washington/40964/2014 (H5N2) HPAIV inoculated surf scoters. A) Mean virus titer equivalents detected by real-time qRT-PCR in OP (closed circles) and CL (open circles) swabs collected from NP/WA/14 (H5N2) inoculated surf scoters at 2, 4, 7, 10, and 14 dpi. B) Area under the curve analyses of total virus equivalents shed from OP and CL routes throughout the duration of the study. Days 0, 2 and 4 post inoculation n=9; days 7, 10 and 14 post inoculation n=8. Error bars represent mean±95% CI. *****p*<0.0001, ***p*<0.01, **p*<0.05.
