# Supplementary material for: The pathogenesis of a North American H5N2 clade 2.3.4.4 group A highly pathogenic avian influenza virus in surf scoters (Melanitta perspicillata)
Source: BMC Vet Res. 2020 Sep 23;16:351. doi: 10.1186/s12917-020-02579-x (PMC7513502; doi:10.1186/s12917-020-02579-x)
Supplement: Supplementary file 3 — Additional file 3. Microscopic lesions and viral antigen distribution in tissues from surf scoters intrachoanally inoculated with A/Northern pintail/Washington/40964/2014 (H5N2) and sampled at 5 (bird #629) and 12 dpi (bird #635). [file 12917_2020_2579_MOESM3_ESM.docx]

**Additional File 3.** Microscopic lesions and viral antigen distribution in tissues from scoters intrachoanally inoculated with H5N8 HPAIV and sampled at 5 (bird #629) and 12 dpi (bird #635).

| **Tissue** | **Histo score^a,b^** | **Lesions** | **IHC score^a,c^** | **Cell types expressing influenza virus antigen** |
| --- | --- | --- | --- | --- |
| Trachea | ++/+ | Focal necrosis with mild lymphocytic inflammatory infiltrate | +/- | Pseudostratified epithelial cells, mononuclear cells, necrotic debris |
| Lung | +/+ | Mild congestion, monocytic infiltrate | -/- | NA^d^ |
| Heart | +/- | Focal necrosis of myocytes | +/- | Myocytes |
| Brain | ++/- | Neuronal necrosis, gliosis, congestion | ++/- | Neurons, ependymal and glial cells |
| Intestine | ++/++ | Moderate lymphohistiocytic enteritis | -/- | NA |
| Pancreas | +++/++ | Moderate to severe multifocal necrosis, congestion, fibrin deposition. Basophilic inclusion bodies in acinar cells | ++/- | Pancreatic acinar cells and mononuclear cells |
| Liver | +++/+++ | Severe multifocal necrosis, congestion, fibrin deposition. Basophilic inclusion bodies in hepatocytes | -/- | NA |
| Spleen | +++/+++ | Severe multifocal necrosis, congestion, fibrin deposition. Basophilic inclusion bodies in reticular cells | -/- | NA |
| Kidney | -/+ | Focal necrosis of tubular epithelium | -/- | NA |
| Proventri-culus | +/++ | Focal to multifocal areas of epithelial necrosis | +/- | Epithelial cells |
| Skeletal Muscle | -/- | NA | -/- | NA |

a. Tissues collected from 2 birds (bird # 629/bird #635).

b. Histopathology score: HE, histologic lesions: - = no lesions; + = mild; ++ = moderate; +++ = severe.

c. IHC = immunohistochemical staining: - = no antigen staining; + = infrequent; ++ = common; +++ = widespread.

d. NA = Not applicable
